# Effects of ketamine and midazolam on resting state connectivity and comparison with ENIGMA connectivity deficit patterns in schizophrenia

**DOI:** 10.1002/hbm.24838

**Published:** 2019-10-21

**Authors:** Bhim M. Adhikari, Juergen Dukart, Joerg F. Hipp, Anna Forsyth, Rebecca McMillan, Suresh D. Muthukumaraswamy, Meghann C. Ryan, L. Elliot Hong, Simon B. Eickhoff, Neda Jahandshad, Paul M. Thompson, Laura M. Rowland, Peter Kochunov

**Affiliations:** ^1^ Maryland Psychiatric Research Center, Department of Psychiatry University of Maryland School of Medicine Baltimore Maryland; ^2^ F. Hoffmann‐La Roche, Pharma Research Early Development Roche Innovation Centre Basel Basel Switzerland; ^3^ Institute of Neuroscience and Medicine, Brain & Behaviour (INM‐7) Research Centre Jülich Jülich Germany; ^4^ Institute of Systems Neuroscience, Medical Faculty Heinrich Heine University Düsseldorf Düsseldorf Germany; ^5^ School of Pharmacy, Faculty of Medical and Health Sciences The University of Auckland Auckland New Zealand; ^6^ Imaging Genetics Center, Mark & Mary Stevens Neuroimaging & Informatics Institute, Keck School of Medicine University of Southern California Marina del Rey California

**Keywords:** effect size, regional homogeneity, resting‐state functional connectivity

## Abstract

Subanesthetic administration of ketamine is a pharmacological model to elicit positive and negative symptoms of psychosis in healthy volunteers. We used resting‐state pharmacological functional MRI (rsPhfMRI) to identify cerebral networks affected by ketamine and compared them to the functional connectivity (FC) in schizophrenia. Ketamine can produce sedation and we contrasted its effects with the effects of the anxiolytic drug midazolam. Thirty healthy male volunteers (age = 19–37 years) underwent a randomized, three‐way, cross‐over study consisting of three imaging sessions, with 48 hr between sessions. A session consisted of a control period followed by infusion of placebo or ketamine or midazolam. The ENIGMA rsfMRI pipeline was used to derive two long‐distance (seed‐based and dual‐regression) and one local (regional homogeneity, ReHo) FC measures. Ketamine induced significant reductions in the connectivity of the salience network (Cohen's *d*: 1.13 ± 0.28, *p* = 4.0 **×** 10^−3^), auditory network (*d*: 0.67 ± 0.26, *p* = .04) and default mode network (DMN, *d*: 0.63 ± 0.26, *p* = .05). Midazolam significantly reduced connectivity in the DMN (*d*: 0.77 ± 0.27, *p* = .03). The effect sizes for ketamine for resting networks showed a positive correlation (*r* = .59, *p* = .07) with the effect sizes for schizophrenia‐related deficits derived from ENIGMA's study of 261 patients and 327 controls. Effect sizes for midazolam were not correlated with the schizophrenia pattern (*r* = −.17, *p* = .65). The subtraction of ketamine and midazolam patterns showed a significant positive correlation with the pattern of schizophrenia deficits (*r* = .68, *p* = .03). RsPhfMRI reliably detected the shared and divergent pharmacological actions of ketamine and midazolam on cerebral networks. The pattern of disconnectivity produced by ketamine was positively correlated with the pattern of connectivity deficits observed in schizophrenia, suggesting a brain functional basis for previously poorly understood effects of the drug.

## 1 INTRODUCTION

Resting‐state pharmacological functional MRI (rsPhfMRI) experiments can map pharmacological effects on brain functional networks by evaluating the time‐course of activity across brain regions and derive coherent spatiotemporal patterns (Biswal, Yetkin, Haughton, & Hyde, 1995; Fox & Raichle, 2007; Margulies et al., 2007; Smith et al., 2009). Here, we used rsPhfMRI to map within‐network connectivity changes induced by ketamine and compared them to the pattern of patient‐control differences observed in schizophrenia and contrast them with the effects of a sedative drug, midazolam (Adhikari et al., 2019). Ketamine and midazolam are routinely used in clinical practice as sedatives though they act through different receptor systems (Allerton, 2009; Green, Rothrock, Lynch, et al., 1998; Green, Rothrock, Harris, et al., 1998). Ketamine is a nonselective antagonist of the glutamate *N*‐methyl‐*d*‐aspartate receptor (NMDAR) while midazolam is a short‐acting benzodiazepine, a positive allosteric modulator of the gamma‐aminobutyric acid (GABA_A_) receptor (Gharde, Chauhan, & Kiran, 2006; Krystal et al., 1994; Morgan & Curran, 2012; Young et al., 2005). A subanesthetic dose of ketamine can elicit positive and negative schizophrenia‐like symptoms in healthy volunteers symptoms (Krystal et al., 1994; Rowland et al., 2005) and in remitted schizophrenia patients (Lahti, Holcomb, Medoff, & Tamminga, 1995). Administration of ketamine in a similar dose has also been approved as a novel therapeutic agent for treatment‐resistant major depression (Abdallah, Averill, & Krystal, 2015; Feder et al., 2014; Williams et al., 2018).

The effects of ketamine include dissociation, disorganized speech, hallucinations, and other psychotic symptoms. This observation suggested that NMDA‐R signaling may play a key role in the pathology of schizophrenia (Lahti et al., 1995). Investigating ketamine effects in healthy volunteers may, therefore, be useful to describe neurophysiological mechanisms associated with schizophrenia‐like symptoms and ketamine has been proposed as a pharmacological model of schizophrenia (Becker et al., 2003; Corlett, Honey, & Fletcher, 2007; Frohlich & Van Horn, 2014). Despite the widespread use of ketamine to model schizophrenia symptoms and a novel treatment for depression, there is very little data on the effects of ketamine on cerebral connectivity and no direct comparisons between its effects and the disconnectivity patterns in schizophrenia. In this manuscript, we evaluate effects of ketamine and midazolam on cerebral connectivity, contrast their effects on cerebral networks and correlate their effects with the ranking of the patient‐control differences in resting‐state functional connectivity (rsFC) a large multi‐cohort sample of schizophrenia patients and controls (Adhikari et al., 2019).

We used two‐long distance metrics and one regional rsFC quantification approach developed by the ENIGMA consortium (Adhikari et al., 2018b). Alterations in the long‐distance connectivity were quantified using seed‐based and dual regression analyses. The seed‐based analysis quantifies rsFC as a correlation of the average BOLD signal time courses between two or more “seed” regions. In the dual regression approach, the group‐average time series for the regions was regressed from individual subjects and whole‐brain correlation maps were computed corresponding to each seed region, to extract the FC values among distant regions. This method provides a statistical inference regarding regional connectivity strengths after correcting for the group average trend. Changes in regional coherence were quantified using the regional homogeneity (ReHo) measure (Zang, Jiang, Lu, He, & Tian, 2004). ReHo measures the FC at a local spatial scale to quantify functional interactions or synchronizations among neighboring voxels. It differs from the other two methods as it maps local spontaneous activity by performing a nearest neighbor analysis of similarity of the BOLD time series (Zuo et al., 2013; Zuo & Xing, 2014). This provides a metric of network centrality to characterize the importance of synchronization in local neuronal networks in the functional connectome.

In this study, we used the placebo as a control and hypothesized that ketamine and midazolam would have both similar and diverging pharmacological effects; and shared and distinct mechanisms of action that could be detected and quantified using rsFC. As both drugs produce sedation, the contrast between them may emphasize networks involved in forming schizophrenia‐like disconnectivity.

## 2 MATERIALS AND METHODS

### 2.1 Study subjects

Thirty healthy male volunteers (average age: 27.3 ± 6.2 years, range: 19–37 years) underwent three pharmacological resting‐state functional imaging scanning sessions. A placebo‐controlled, randomized, three‐way cross‐over design was used where participants were blind to the drug they were receiving. Each subject participated in all three sessions. Individual sessions were separated by 48 hr to allow for a wash‐out of drug effects. Each session collected resting‐state imaging data. Participants were instructed to have their eyes open and fixated on a small cross on a projection screen during infusion. Written informed consent was obtained from all participants and the local Ethics Committee approved the study. The data analyzed here have been previously reported in (Forsyth et al., 2018; McMillan et al., 2019).

### 2.2 Imaging protocol

All experiments were conducted using a 3 T MRI scanner (Siemens Skyra, Erlangen, Germany) equipped with a 20‐channel head coil. BOLD fMRI data were acquired using a T2*‐weighted echo‐planar imaging (EPI) sequence (repetition time (TR) 2,200 ms, echo time (TE) 27 ms, flip angle 79°, 30 interleaved 3‐mm slices, field of view 215 mm, voxel size 3 **×** 3 **×** 3 mm^3^). For the resting‐state data reported here, 437 volumes were acquired (7 min predrug, 9 min postdrug).

Drugs were administered to a subanesthetic level through an intravenous line controlled by an infusion pump (Alaris PK, UK), programmed by a supervising anesthesiologist, located in the MR control room. Ketamine was administered with a 0.25 mg/kg bolus dose, followed by a 0.25 mg/kg/hr infusion. Doses were similar to those used in previous literature (Deakin et al., 2008; Muthukumaraswamy et al., 2015). Midazolam was administered with a 0.03 mg/kg bolus dose, followed by a 0.03 mg/kg/hr infusion, resulting in doses similar to prior studies (Greicius et al., 2008; Liang et al., 2015). Participants were monitored for their respiration and blood pressure during data acquisition (as detailed in the Supporting Information). Participants were asked if they fell asleep postscanning and none of them reported to have fallen asleep. All participants performed alertness tasks after rsfMRI scanning to ensure that they did not fall asleep during data collection.

### 2.3 Resting‐state functional MRI data processing and analysis

Prior to preprocessing, data for each condition were split into the first and last 190 volumes (7 min), resulting in predrug and postdrug administration datasets and removal of the 2‐min bolus period. The ENIGMA resting‐state analysis pipeline implemented in the Analysis of Functional NeuroImages (AFNI) software (Cox, 1996) was used to process the rsfMRI data. This single‐modality resting‐state analysis pipeline (Adhikari et al., 2018) is an extension of the conventional AFNI rsfMRI pipeline (Figure S1). A principal component analysis (PCA)‐based denoising (Veraart, Novikov, et al., 2016; Veraart, Fieremans, & Novikov, 2016) approach is the first step implemented in this analysis pipeline to improve signal‐to‐noise ratio (SNR) and temporal SNR (tSNR) properties of the time series data. This denoising approach called Marchenko–Pastur (MP)‐PCA (MPPCA), neither alters the spatial resolution of the image nor introduces additional partial volume effects that can lead to complications in further quantitative analyses (Veraart, Novikov, et al., 2016). In the next step, a transformation is computed registering the base volume to the ENIGMA EPI template derived from ~1,100 datasets collected across 22 sites (Adhikari, Jahanshad, Shukla, Turner, et al., 2018) and this atlas is used for regression of the global signal, and as a common anatomical spatial reference frame. RsfMRI data processing is detailed in the Supporting Information.

Resting‐state network templates were defined based on the probabilistic regions of interest (ROIs) from an independent components analysis of the BrainMap activation database and resting‐state fMRI dataset (Smith et al., 2009). Binary masks of the resting state template regions were defined for the auditory network (AN), attention network (AttN), default mode network (DMN), executive‐control network (ECN), frontoparietal network (FPN), salience network (SN), sensorimotor network (SMN), and visual network (VN; Figure S2; Adhikari et al., 2018aa). Resting‐state functional connectivity (rsFC) values between functional connections were extracted from these template ROIs using seed‐based and dual regression analysis approaches (as detailed in the Supporting Information) and the subsequent analysis was performed using these measures.

Regional homogeneity (ReHo) was designed to investigate changes in local spontaneous brain activity by performing a nearest neighbor analysis of similarity of the BOLD time series and assigning a Kendall's coefficient of concordance (KCC) score (Zang et al., 2004). Equation (1) shows how the KCC score is calculated per voxel based on signals from neighboring voxels:

(1) $$W=\frac{\sum \left(R_{i}^{2}\right)-nR^{2}}{\frac{1}{12}K^{2}\left(n^{3}-n\right)}$$

Here *W* is the KCC among given voxels, ranging from 0 to 1; *R*_*i*_ is the sum rank of the *i*th time point; *R* = ([*n* + 1] *K*)/2 is the mean of the *R*_*i*_ *'*s; *K* is the number of time series within a measured cluster (*K* is set to be 7 [for facewise neighbors only], 19 [for face‐ and edge‐wise neighbors], or 27 [for face‐, edge‐, and node‐wise neighbors]), that is, one given voxel plus a number of its neighbors, and *n* is the number of ranks (here, *n* = 190; Zang et al., 2004).

To optimize the trade‐off between mitigation of partial volume effects and generation of Gaussian random fields, we set *K* = 27, as per recommendations, to cover all directions in 3D space (Jiang & Zuo, 2016). This score offers advantages for three different aspects of measuring local functional connectivity: (*i*) the nearest neighboring nodes can be defined spatially, which usually reflects the anatomical, morphological, and geometric features in a local brain structure; (*ii*) the rank‐based computation is efficient in the time domain; and (*iii*) this score is robust against noise by integrating noise‐filtering operations across both the spatial domain and the temporal domain. Of note, the ReHo values of the voxels near the border of the brain mask need to be discussed with caution. The ReHo map, the collection of all voxels' KCC score of each voxel with (typically) 26 nearest neighboring voxels, for each subject each condition, was calculated using the MATLAB function “*y_reho.m”* available in the DPABI_V3.0_171210 package. The ReHo calculation was performed in three‐dimensional volumetric space, and still the calculation on the two‐dimensional cortical surface is preferred for the better representation of the neural neighborhood across the heavily folded cortex (Bijsterbosch, Smith, & Beckmann, 2017). Moreover, the ReHo is less sensitive to potential differences in the shape of the local neighborhood; the size and shape of functional regions in the brain are not uniform and require a careful interpretation of these measures in the light of other long‐distance FC measures.

KCC scores were extracted from the resting state networks' ROIs and subsequent analyses were performed using these measures.

### 2.4 Analysis of connectivity measures and regional homogeneity KCC scores

Paired‐sample *t*‐tests were performed between the postdrug (postinfusion) and predrug (preinfusion) rsFC measures from both the seed‐based and dual regression analysis approach for the ketamine, midazolam, and placebo conditions. We compared KCC scores from each of the resting state template ROIs between postinfusion and preinfusion of the drugs and the placebo.

The difference between postinfusion and preinfusion measures (expressed in terms of Cohen's *d*‐values)—for ketamine, midazolam and the placebo—was calculated for each of the resting‐state functional connections; and for seed‐based and dual regression analysis approaches, separately. Similarly, Cohen's *d*‐values (postinfusion relative to preinfusion) were calculated using KCC scores for each of the resting state template ROIs.

To compare the measurements obtained from the three analysis approaches, we calculated mean *d*‐values for the functional connections within the RSN both for seed‐based and dual regression analysis approaches, and a mean KCC score for each RSNs. We further divided the AttN and FPN into left and right networks each (lAttN, rAttN and lFPN, rFPN), keeping the ROIs and the functional connections within the same hemisphere. The reasons behind this are that the FPN exhibited strong within‐hemisphere interactions but with distinct patterns in each hemisphere and preferentially coupled to the DMN and language‐related regions in the left hemisphere but to AttN in the right hemisphere (Spreng, Stevens, Chamberlain, Gilmore, & Schacter, 2010; Vincent, Kahn, Snyder, Raichle, & Buckner, 2008; Wang, Buckner, & Liu, 2014). Also, studies examining functional connectivity have shown that FPN is asymmetrically organized (Habas et al., 2009).

To determine the drug impacts beyond the placebo effect, we compared (paired‐sample *t* tests) the rsFC measures derived from RSNs, and KCC scores from ReHo methods between the postinfusion minus preinfusion for the drugs and the postinfusion minus preinfusion for the placebo.

### 2.5 Effects of ketamine and midazolam and network‐based patient‐control differences in schizophrenia

We performed a correlation analysis between spatial maps of the effect sizes of ketamine and midazolam versus patient‐control differences in the connectivity of the RSNs in schizophrenia. The rank effect sizes of schizophrenia on the networks were evaluated by a large mega‐and‐meta analytic study and shown in Table S3 (Adhikari et al., 2019). We performed a correlation analysis of the effect sizes in schizophrenia with those calculated for ketamine, midazolam, and ketamine–midazolam contrasts from both long‐distance functional connectivity analysis approaches.

## 3 RESULTS

### 3.1 Effect of drugs on the seed‐based rsFC measures

**Figure 1:**
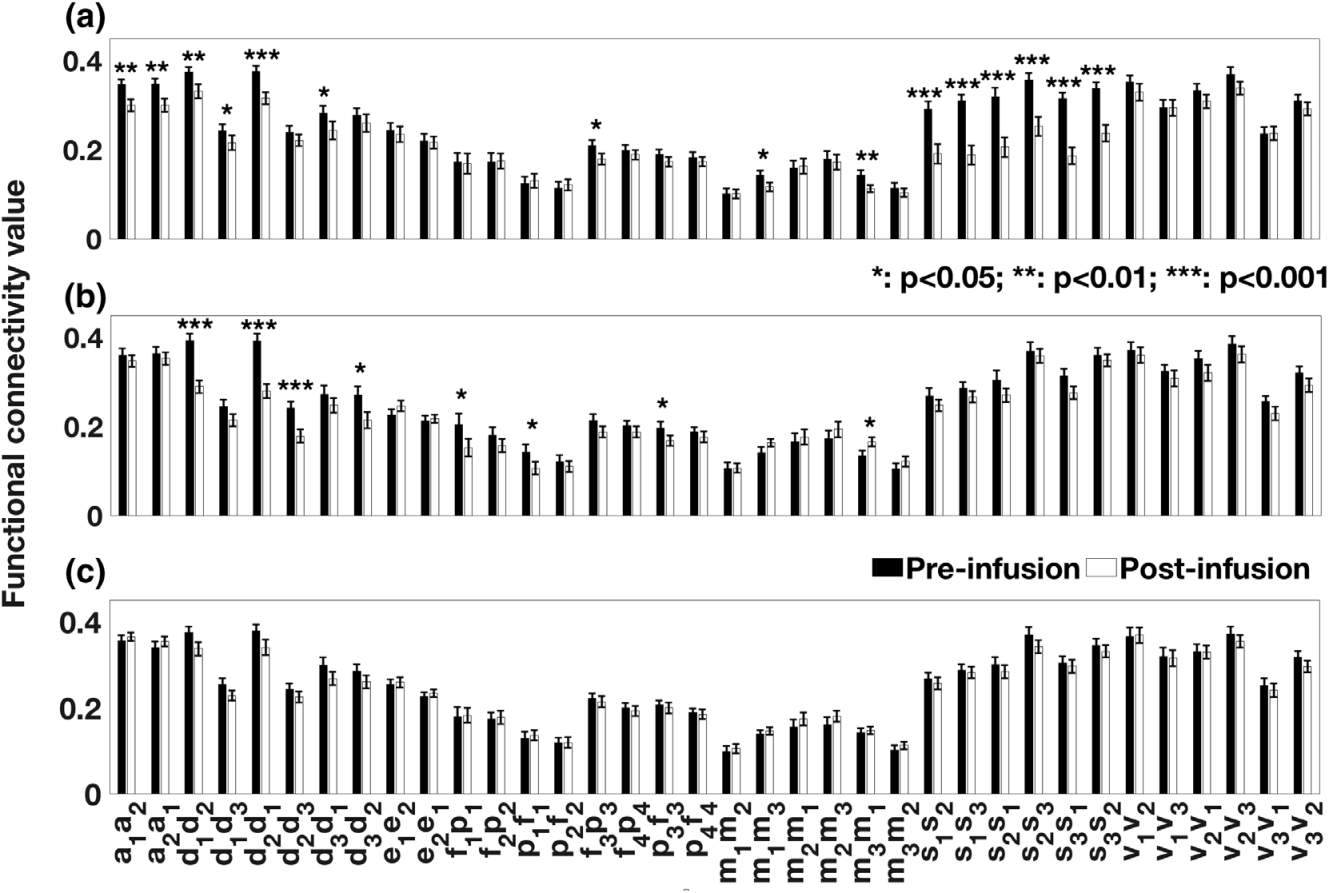
Comparison of resting‐state functional connectivity strengths, postinfusion relative to preinfusion, for (a) ketamine, (b) midazolam, and (c) placebo in seed‐based analysis approach. The regions of interest (ROIs) are based on Figure S2. ROIs are: a_1_/a_2_, left/right primary and association auditory cortices (auditory network); f_1_/f_2_, left/right middle frontal gyrus, p_1_/p_2_, left/right superior parietal lobule (attention network); d_1_, posterior cingulate/precuneus, d_2_, bilateral temporal–parietal regions, d_3_, ventromedial frontal cortex (default mode network); e_1_, anterior cingulate cortex, e_2_, bilateral medial frontal gyrus (executive–control network); f_3_/f_4_, left/right inferior frontal gyrus, p_3_/p_4_, left/right inferior parietal lobule (frontoparietal network); s_1_, anterior cingulate cortex, s_2_/s_3_, left/right insula (salience network); m_1_/m_3_, left/right motor area, m_2_, supplementary motor area (sensorimotor network); v_1_, medial visual areas, v_2_, occipital visual areas, and v_3_, lateral visual areas (visual network). Functional connections are defined from one ROI to another ROI within the resting state network (e.g., a_1_a_2_ represents for the functional connection from a_1_ to a_2_)

Ketamine administration led to a significant reduction in the connectivity strength in the salience network (SN, Cohen's *d*: 1.13 ± 0.28) followed by the auditory network (AN, *d*: 0.67 ± 0.26) and default mode network (DMN, *d*: 0.63 ± 0.26; Figure 1a, Table 1, Table S2). For midazolam, the strongest effect was observed in the DMN (*d*: 0.77 ± 0.27) followed by the left attention network (lAttN, *d*: 0.43 ± 0.26) and the left frontoparietal network (lFPN, *d*: 0.39 ± 0.26). Other connections showing significantly reduced FC strength under midazolam were between the left middle frontal area and the left superior parietal area (attention network, AttN); and from the left inferior parietal lobule to the left inferior frontal gyrus (frontoparietal network, FPN). The only functional connection that showed a significant increase in FC was from the left motor cortex to right motor cortex in SMN (*p* < .05) (Figure 1a, Table 1). Placebo (saline) did not alter the rsFC strengths (Table 1).

**Table 1:** Statistical measures (*p*‐value and *t*‐value) for the comparison between functional connectivity measures derived from resting‐state networks (RSNs) using seed‐based analysis approach for resting scans during postinfusion and preinfusion of drugs: ketamine and midazolam and placebo

Network	Connections	Ketamine	Midazolam	Placebo
		*p* (*t*)	*p* (*t*)	*p* (*t*)
Auditory network (AN)	Auditory cortices L–auditory cortices R	**6.0 × 10** ^**−3**^ **(−2.99)**	0.38 (−0.90)	0.29 (1.08)
	Auditory cortices R–auditory cortices L	**1.2 × 10** ^**−3**^ **(−3.76)** a	0.51 (−0.67)	0.17 (1.40)
Attention network (AttN)	Middle FG L–SPL L	0.80 (−0.26)	**2.2 × 10** ^**−2**^ **(−2.44)**	0.82 (0.23)
	SPL L–middle FG L	0.54 (0.62)	**2.5 × 10** ^**−2**^ **(−2.38)**	0.60 (0.53)
	Middle FG R–SPL R	0.88 (0.15)	0.13 (−1.57)	0.79 (0.27)
	SPL R–middle FG R	0.51 (0.67)	0.29 (−1.07)	0.91 (0.11)
Default mode network (DMN)	PCC/precuneus–bilateral temporal–parietal region	**1.0 × 10** ^**−3**^ **(−3.82)** ^a^	**2.1 × 10** ^**−7**^ **(−6.89)** a	0.10 (−1.65)
	PCC/precuneus–vmFC	**1.3 × 10** ^**−3**^ **(−3.69)** a	0.06 (−1.98)	0.06 (−1.93)
	Bilateral temporal–parietal region–PCC/precuneus	**3.7 × 10** ^**−4**^ **(−4.05)** a	**1.1 × 10** ^**−6**^ **(−6.26)** a	0.13 (−1.50)
	Bilateral temporal–parietal region–vmFC	0.27 (−1.12)	**3.5 × 10** ^**−4**^ **(−4.09)** a	0.22 (−1.26)
	vmFC–PCC/precuneus	**1.1 × 10** ^**−2**^ **(−2.72)**	0.21 (−1.26)	0.06 (−1.98)
	vmFC–bilateral temporal–parietal region	0.32 (−1.01)	**1.3 × 10** ^**−2**^ **(−2.67)**	0.09 (−1.75)
Executive control network (ECN)	ACC–bilateral medial FG	0.65 (−0.46)	0.14 (1.51)	0.74 (0.33)
	Bilateral medial FG–ACC	0.84 (−0.20)	0.61 (0.52)	0.57 (0.58)
Fronto‐parietal network (FPN)	IFG L–IPL L	**3.0 × 10** ^**−3**^ **(−3.29)**	0.07 (−1.91)	0.40 (−0.86)
	IPL L–IFG L	0.11 (−1.63)	**2.0 × 10** ^**−2**^ **(−2.47)**	0.42 (−0.83)
	IFG R–IPL R	0.42 (−0.81)	0.20 (1.31)	0.45 (−0.76)
	IPL R–IFG R	0.38 (−0.89)	0.25 (−1.19)	0.89 (−0.56)
Salience network (SN)	ACC–INS L	**5.3 × 10** ^**−5**^ **(−4.76)** a	0.18 (−1.38)	0.51 (−0.66)
	ACC–INS R	**5.0 × 10** ^**−6**^ **(−5.63)** a	0.36 (−0.93)	0.75 (−0.32)
	INS L–ACC	**2.0 × 10** ^**−5**^ **(−5.12)** a	0.06 (−1.99)	0.26 (−1.14)
	INS L–INS R	**5.7 × 10** ^**−6**^ **(−5.58)** a	0.65 (−0.46)	0.09 (−1.76)
	INS R–ACC	**1.3 × 10** ^**−6**^ **(−6.12)** a	0.06 (−1.99)	0.67 (−0.44)
	INS R–INS L	**1.1 × 10** ^**−5**^ **(−5.33)** a	0.60 (−0.53)	0.32 (−1.02)
Sensorimotor network (SMN)	Motor area L–SMA	0.98 (−0.03)	0.99 (0.00)	0.42 (0.82)
	Motor area L–motor area R	**2.1 × 10** ^**−2**^ **(−2.46)**	0.09 (1.74)	0.45 (0.76)
	SMA–motor area L	0.81 (0.24)	0.65 (0.47)	0.16 (1.44)
	SMA–motor area R	0.72 (−0.37)	0.34 (0.97)	0.57 (1.99)
	Motor area R–motor area L	**5.0 × 10** ^**−3**^ **(−3.02)**	**2.8 × 10** ^**−2**^ **(2.33)**	0.52 (0.65)
	Motor area R–SMA	0.37 (−0.92)	0.28 (1.10)	0.14 (1.52)
Visual network (VN)	Medial visual areas–occipital visual areas	0.21 (−1.27)	0.65 (−0.46)	0.86 (0.18)
	Medial visual areas–lateral visual areas	0.99 (0.01)	0.50 (−0.69)	0.84 (−0.21)
	Occipital visual areas–medial visual areas	0.13 (−1.54)	0.17 (−1.40)	0.99 (−0.02)
	Occipital visual areas–lateral visual areas	0.07 (−1.95)	0.24 (−1.19)	0.31 (−1.03)
	Lateral visual areas–medial visual areas	0.95 (0.06)	0.20 (−1.32)	0.49 (−0.70)
	Lateral visual areas–occipital visual areas	0.11 (−1.64)	0.14 (−1.53)	0.13 (−1.54)

Note: Bolded figures are significant for *p* < .05. The regions are based on Figure S2.

Abbreviations: ACC, anterior cingulate cortex; FG, frontal gyrus; IFG, inferior frontal gyrus; INS, insula; IPL, inferior parietal lobule; L, left; PCC, posterior cingulate cortex; R, right; SMA, supplementary motor area; SPL, superior parietal lobule; vmFC, ventromedial frontal cortex.

a Values those are significant after correction for multiple comparisons.

### 3.2 Effect of drugs on the dual‐regression rsFC measures

Effects of ketamine and midazolam in the dual regression analysis were very similar to those from the seed‐based analyses (Figure S3, Table S1). There were also no significant placebo effects.

### 3.3 Effect of drugs on the ReHo rsFC measures

**Figure 2:**
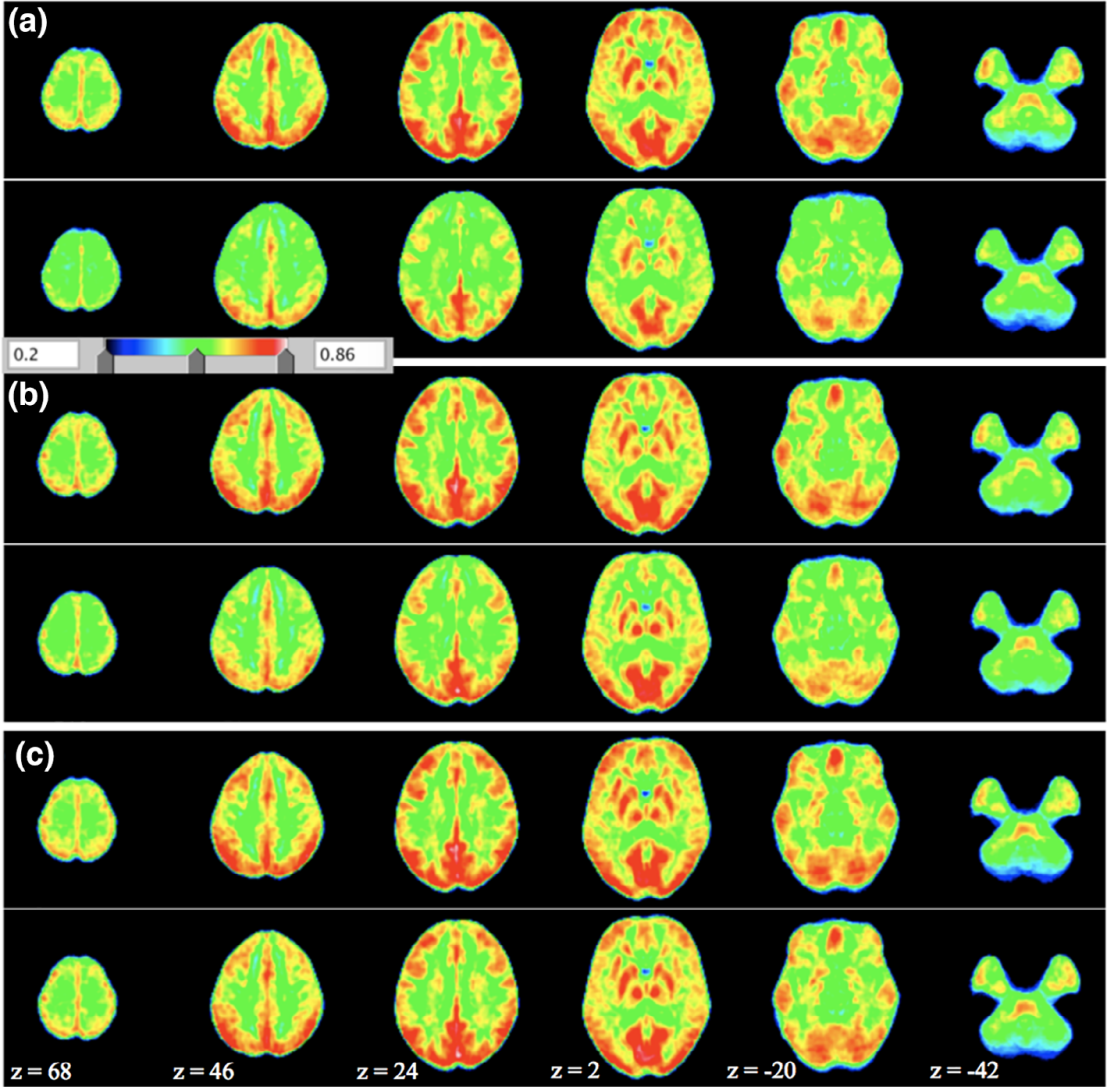
Average ReHo maps for (a) ketamine, (b) midazolam, and (c) placebo condition. In each subplot (a–c), the upper panel shows the pre‐infusion and lower panel shows the postinfusion. Shown are the ReHo maps (with parameters combination of spatial smoothing FWHM, full width at half maximum, 0 mm and cluster size, 27). The z‐coordinates of the displayed axial slices are provided

**Figure 3:**
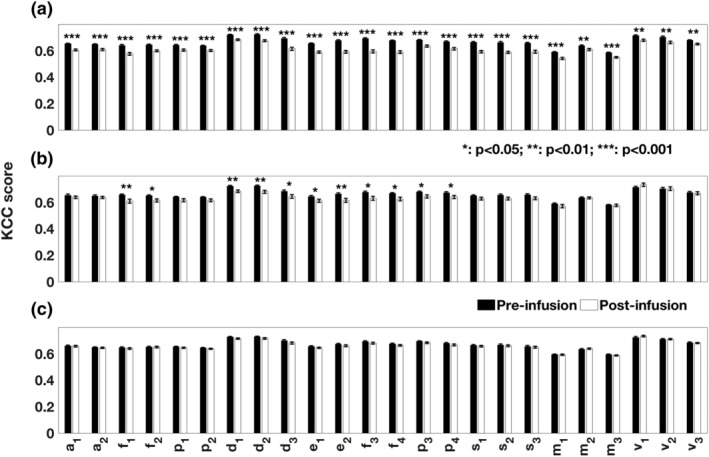
Comparison of Kendall's coefficients of concordance (KCC) obtained from the resting state networks ROIs for (a) ketamine, (b) midazolam, and (c) placebo condition (postinfusion relative to vs preinfusion). The names and the notations of ROIs are based on Figure 1

Ketamine significantly reduced ReHo scores for all the RSNs ROIs (all *p <* .01; Figures 2 and 3 and Table 2). Effects of midazolam were limited to significantly decreased ReHo KCC scores for ROIs of the DMN, the ECN, the FPN and the frontal areas of the AttN (*p* < .05; Figures 2, 3, and Table 2). There were no significant changes for placebo.

**Table 2:** Statistical measures (*p*‐value and *t*‐value) for the comparison between regional homogeneity measures (Kendall's coefficient of concordance, KCC) derived from RSNs ROIs for postinfusion and preinfusion of drugs: ketamine and midazolam and the placebo condition. Here, preinfusion means predrug/placebo condition and postinfusion means postdrug/placebo condition

Network	Regions of interest (ROIs)	Ketamine	Midazolam	Placebo
		*p* (*t*)	*p* (*t*)	*p* (*t*)
Auditory network (AN)	Auditory cortices L	**9.9 × 10** ^**−6**^ **(−4.86)** ^a^	0.30 (−1.06)	0.74 (−0.33)
	Auditory cortices R	**3.7 × 10** ^**−4**^ **(−3.79)** a	0.36 (−0.92)	0.72 (−0.36)
Attention network (AttN)	Middle FG L	**3.4 × 10** ^**−6**^ **(−5.16)** a	**2.0 × 10** ^**−3**^ **(−3.26)** a	0.52 (−0.64)
	SPL L	**7.8 × 10** ^**−4**^ **(−3.55)** a	0.08 (−1.78)	0.39 (−0.87)
	Middle FG R	**4.0 × 10** ^**−5**^ **(−4.46)** a	**1.4 × 10** ^**−2**^ **(−2.54)**	0.98 (−0.03)
	SPL R	**5.0 × 10** ^**−3**^ **(−2.91)**	0.09 (−1.71)	0.27 (−1.12)
Default mode network (DMN)	PCC/precuneus	**3.3 × 10** ^**−5**^ **(−4.51)** a	**2.1 × 10** ^**−4**^ **(−3.97)** a	0.10 (−1.67)
	Bilateral temporal–parietal region	**2.2 × 10** ^**−5**^ **(−4.63)** a	**2.0 × 10** ^**−3**^ **(−3.26)** a	0.14 (−1.50)
	vmFC	**2.6 × 10** ^**−6**^ **(−5.23)** a	**2.7 × 10** ^**−2**^ **(−2.28)**	0.19 (−1.33)
Executive control network (ECN)	ACC	**4.0 × 10** ^**−8**^ **(−6.35)** a	**2.5 × 10** ^**−2**^ **(−2.31)**	0.25 (−1.17)
	Bilateral medial FG	**3.9 × 10** ^**−8**^ **(−6.36)** a	**1.8 × 10** ^**−3**^ **(−3.28)** a	0.20 (−1.31)
Fronto‐parietal network (FPN)	IFG L	**1.7 × 10** ^**−5**^ **(−6.58)** a	**1.3 × 10** ^**−2**^ **(−2.57)**	0.24 (−1.18)
	IPL L	**5.8 × 10** ^**−5**^ **(−4.35)** ^a^	**1.8 × 10** ^**−2**^ **(−2.45)**	0.23 (−1.22)
	IFG R	**9.9 × 10** ^**−9**^ **(−6.72)** a	**1.6 × 10** ^**−2**^ **(−2.48)**	0.26 (−1.14)
	IPL R	**1.6 × 10** ^**−5**^ **(−4.72)** a	**3.7 × 10** ^**−2**^ **(−2.14)**	0.25 (−1.16)
Salience network (SN)	ACC	**3.1 × 10** ^**−8**^ **(−6.43)** a	0.10 (−1.65)	0.41 (−0.83)
	INS L	**1.7 × 10** ^**−8**^ **(−6.59)** a	0.09 (−1.74)	0.59 (−0.55)
	INS R	**7.2 × 10** ^**−5**^ **(−4.29)** a	0.06 (−1.96)	0.64 (−0.48)
Sensorimotor network (SMN)	Motor area L	**2.5 × 10** ^**−5**^ **(−4.59)** a	0.22 (−1.25)	0.93 (−0.08)
	SMA	**1.2 × 10** ^**−2**^ **(−2.59)**	0.99 (0.00)	0.49 (0.70)
	Motor area R	**4.1 × 10** ^**−5**^ **(−4.45)** a	0.78 (−0.28)	0.28 (−1.09)
Visual network (VN)	Medial visual areas	**2.0 × 10** ^**−3**^ **(−3.25)** a	0.25 (1.18)	0.29 (1.06)
	Occipital visual areas	**1.8 × 10** ^**−3**^ **(−3.27)** a	0.98 (−0.03)	0.87 (0.16)
	Lateral visual areas	**2.7 × 10** ^**−3**^ **(−3.14)**	0.65 (−0.46)	0.70 (−0.38)

Note: Bolded figures are significant for *p* < .05. Regions are based on Figure S2.

Abbreviations: ACC, anterior cingulate cortex; FG, frontal gyrus; IFG, inferior frontal gyrus; INS, insula; IPL, inferior parietal lobule; L, left; PCC, posterior cingulate cortex; R, right; SMA, supplementary motor area; SPL, superior parietal lobule; vmFC, ventromedial frontal cortex.

a Values those are significant after correction for multiple comparisons.

### 3.4 Agreement among rsFC measures

**Figure 4:**
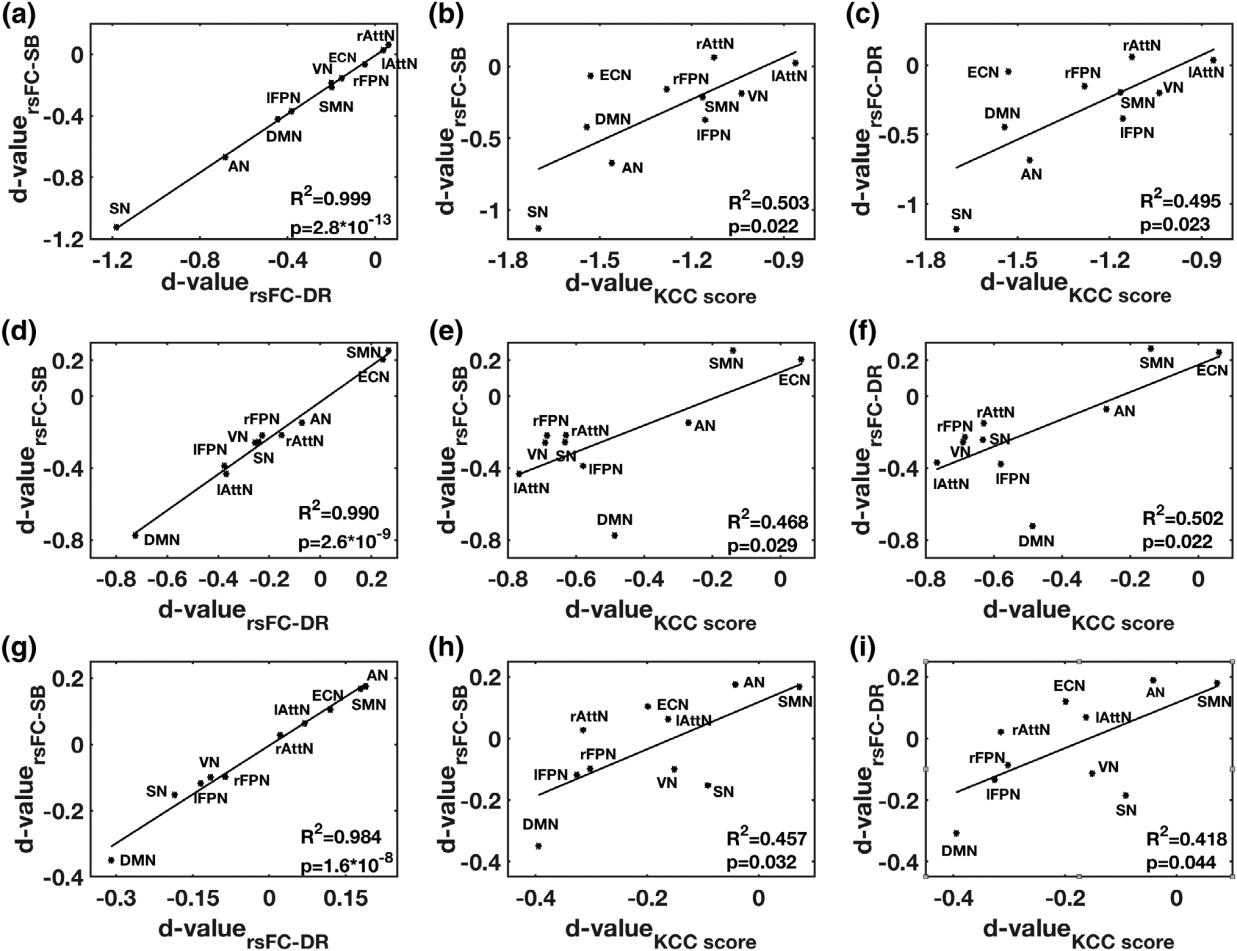
The relationships between Cohen's *d*‐values derived from rsFC measures derived from three methods (SB, DR, and ReHo), from resting‐state networks, are described by *R* ^2^ and *p*‐value in each subplot. The relationships between these measures are shown in first row (a–c) for ketamine, the second row (d–f) for midazolam, the third row (g–i) for placebo and first column for SB and DR approaches, second column for SB and ReHo approaches and third column for DR and ReHo approaches. AN, auditory network, lAttN/rAttN, left/right attention network, DMN, default mode network, ECN, executive‐control network, lFPN/rFPN, left/right frontoparietal network, SMN, sensorimotor network, SN, salience network and VN, visual network, SB, seed‐based, DR, dual regression, KCC, Kendall's coefficient of concordance

Cohen's *d* effect size values for the rsFC measures in the seed‐based and dual regression analysis approaches were highly correlated (*r* = .98, *p* < 10^−7^) for ketamine, midazolam, and the placebo (first column; Figure 4). Significant linear relationships (*r* > .65, *p* < .05) were found between rsFC measures in the seed‐based and dual regression approaches with ReHo KCC scores (Figure 4).

The rsFC measures derived from RSNs showed a significant reduction in FC strength for SN connections (*p* < .001) and AN connections (*p* < .01) with ketamine administration. Significant reductions in FC strengths for the DMN and left AttN (lAttN) connections (*p* < .01) with midazolam (Figure S4) for the comparison between postinfusion minus preinfusion and postinfusion minus preinfusion for placebo condition. All the RSNs ROIs showed significant effects and reduced KCC scores with ketamine (*p* < .01) whereas AN, SN, SMN, and VN ROIs showed no significant changes with midazolam (Figure S5).

### 3.5 Effects of ketamine, midazolam, and their difference versus network‐based patient‐control differences in schizophrenia

**Figure 5:**
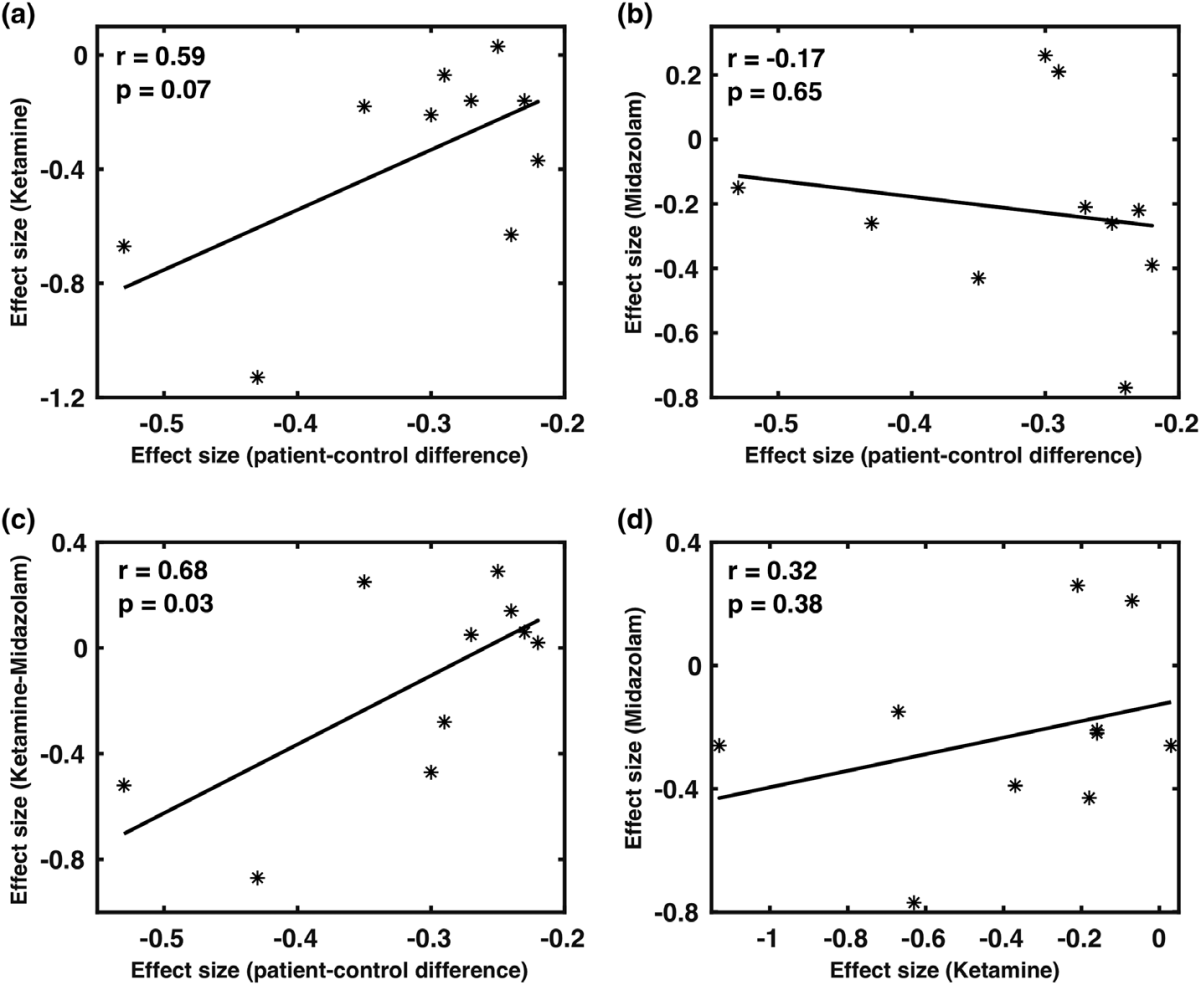
The relationships between effect sizes of ketamine (a), midazolam (b), and their difference (c) versus network‐based patient‐control differences in schizophrenia. Subplot (d) represents the relationship between the effect sizes of midazolam versus the effect sizes of ketamine (positive, but nonsignificant). We observed a significant positive correlation with effect sizes for ketamine–midazolam contrast (c); tending to significant positive correlation for ketamine (a), and no correlation for midazolam (b) with the effect sizes in schizophrenia

The effect sizes of ketamine on the activities of the AN (Cohen's *d* = 0.67) and SN (Cohen's *d* = 1.13) were roughly twice the significant patient‐control differences observed in rsFC measures for SN (Cohen's *d* = 0.43), and AN (Cohen's *d* = 0.53) in schizophrenia (Adhikari et al., 2019). The disconnectivity effect (reduction in rsFC) of ketamine (Cohen's *d* = 0.63) on the DMN connectivity was not significant in the patient‐control differences in schizophrenia (Cohen's *d* = 0.24). The effect sizes of ketamine were found correlated (tending to be significant, *r* = .59, *p* = .07) with the network‐based patient‐control differences in the FC in schizophrenia, but no association was found for midazolam (*r* = −.17, *p* = .65; Figure 5a,b). The subtraction of the ketamine and midazolam was significantly correlated with the effect sizes in schizophrenia (*r* = .68, *p* = .03; Figure 5c). Ketamine and midazolam both showed overlapping effects on rsFC in the DMN and the effect sizes of these drugs across all networks showed a positive but a nonsignificant correlation (*r* = .32, *p* = .38; Figure 5d). Results, using the effect size measures from the dual regression analyses, were similar to those obtained from the seed‐based analyses.

## 4 DISCUSSION

A single‐blind, randomized cross‐over approach was used to investigate the shared and distinct effects of ketamine and midazolam on the activity of brain functional networks and compare them with the pattern of cerebral disconnectivity observed in schizophrenia patients. The effects of the two drugs were readily detected by three independent resting‐state analysis approaches. Administration of a placebo produced no significant changes. Both drugs reduced the activity in the default mode network (DMN) that we attributed to sedation. The effect sizes of ketamine (Cohen's *d* ~ 1.0) on activities of the DMN, SN, and AN were about twice those observed in the patient‐control differences in these networks in schizophrenia and the correlation between the patterns of affected networks approached significance (*r* = .59, *p* = .07; Adhikari et al., 2019). The effects of the midazolam were not correlated with the effects of ketamine or the schizophrenia‐related pattern of disconnectivity. However, the contrast between ketamine and midazolam patterns of disconnectivity was significantly correlated with the pattern in schizophrenia (*r* = .68, *p* = .03), presumably by reducing the sedation effects that both drugs exerted through DMN. Overall, this study suggests that ketamine challenges can produce the pattern of functional disconnectivity that mimics that observed in schizophrenia with exception of sedative effects of ketamine that can be corrected by contrasting with the effects of the midazolam challenge.

### 4.1 Effects of ketamine on rsFC measures

Following ketamine administration, there was a significant reduction in rsFC strength using both the long‐distance (seed‐based and dual regression analysis) and localized (ReHo) measures of connectivity. Overall, these results are consistent with findings from an fMRI study that used ketamine as one of the functional contrasts (Mueller et al., 2018). The observed significant reductions in the AN rsFC for ketamine were consistent with previously reported decreased connectivity in the auditory, visual, and somatosensory network regions in relation to predefined networks of interest (Niesters et al., 2012). Our finding of the significant reductions in rsFC for DMN is in agreement with the previously reported decreased FC strength of the PCC with other DMN regions (Scheidegger et al., 2012) and that of vmFC with other DMN regions with increasing depth of ketamine sedation (Bonhomme et al., 2016). No significant effects of ketamine were observed for the visual or sensorimotor networks, which provide support for ketamine primarily affecting higher‐order integration networks, such as the DMN and SN (Bonhomme et al., 2016). We did not find changes in rsFC for ECN with ketamine, which is in contrast to a recent study that showed a significant positive effect of ketamine on the connectivity in ECN (Mueller et al., 2018). For ketamine administration, we also did not observe any changes in frontoparietal connectivity; even so, ketamine doses high enough to induce loss of responsiveness have caused impaired frontal–parietal connectivity (Blain‐Moraes, Lee, Ku, Noh, & Mashour, 2014; Lee et al., 2013).

### 4.2 Effects of midazolam on rsFC measures

Following midazolam administration, we observed a significant reduction in rsFC strength in the DMN, AttN, and FPN connections using long‐distance connectivity approaches. The ECN regions showed the reduced local rsFC using short‐distance connectivity approach. Midazolam showed no significant changes in the rsFC for lower‐level networks such as AN and VN, hence the connectivity of these networks was found to be intact, consistent with the previous findings (Liang et al., 2015). Midazolam inhibited the higher‐level networks—such as DMN, AttN, and FPN. These results were consistent with prior findings (Heine et al., 2012) that higher‐order brain network interactions were impaired when the consciousness level was reduced. We found midazolam‐related elevation of FC in one functional connection—from right motor cortex to left motor cortex in the SMN—although the SN, the FPN, and language network had shown elevated FC induced by midazolam (Liang et al., 2015). The decreased connectivity in these networks may represent a correlate of reduced consciousness (Boveroux et al., 2010; Greicius et al., 2008; Guldenmund et al., 2013) that may be responsible for the decline in cognitive functions such as episodic memory, executive functions, semantic processing under sedation/anesthesia from drugs.

### 4.3 Similarity and contrast between ketamine and midazolam

Ketamine and midazolam both showed overlapping effects on rsFC in the DMN. The effect sizes of these drugs across all networks showed a positive yet nonsignificant correlation suggesting a distinct action of two drugs in other networks. Ketamine actions extended to the SN and AN, while midazolam affected FPN and AttN. Our findings are consistent with the findings from a double‐blind, placebo‐controlled, cross‐over fMRI study that examined the effects of a single ketamine infusion on FC and reported changes in the DMN, SN, and ECN in patients with major depressive disorder (Evans et al., 2018). A greater reduction in suicidal ideation within 24 hr in depressed patients was found for ketamine compared to midazolam (Grunebaum et al., 2018). The sedative effects of ketamine were weaker than midazolam allowing for quicker recovery (Wilkinson et al., 2019). The reduction in FC in the SN and DMN that we observed after ketamine administration in healthy subjects may have implications for antidepressant treatment (Hamilton et al., 2011) due to the increased level of DMN dominance in depression that associates with higher levels of maladaptive, depressive rumination, and lower levels of adaptive, reflective rumination (Berman et al., 2011; Hamilton et al., 2011). The existing antidepressant medications have been shown to work in part by reducing FC in the DMN and ketamine could be producing the same effect but much faster (Posner et al., 2013; Wang et al., 2015; Williams et al., 2018).

### 4.4 Effects of ketamine, midazolam and their difference versus patient‐control differences in schizophrenia

The significant reduction in effect sizes for the functional connectivity of the AN and SN for ketamine is consistent with the significant patient‐control differences observed in these networks in a large mega‐and‐meta analytic study in schizophrenia (Adhikari et al., 2019; Joules et al., 2015). The marginally significant correlation between the effect sizes of ketamine and the patient‐control differences in schizophrenia was found significant following the subtraction of the effects of midazolam. This step has reduced the sedative effects, shared by the two drugs, and emphasized the effects of NMDAR‐related disconnectivity in higher cognitive networks.

Limitations include the enrollment of male subjects only in rsPhfMRI study, and the lack of ratings to measure psychotomimetic reaction to ketamine and midazolam. Only males were recruited to reduce potential variability of the menstrual cycle during the study, which lasted over 9 days (three imaging sessions with 2 days in between them). We believe this to be a minor limitation because there were no significant sex differences in the schizophrenia pattern of cerebral disconnectivity in schizophrenia (Adhikari et al., 2019; Joules et al., 2015). Furthermore, future research is warranted in an external validation dataset in order to replicate the research findings. The patterns of affected networks in ketamine administration and schizophrenia‐related patterns of disconnectivity approached significance in our sample, which highlights the necessity of carrying out a research study in a larger sample size in order to investigate the reliability of these research findings and to check whether the effects detected are biological.

## 5 CONCLUSION

In healthy male volunteers, both ketamine and midazolam led to significantly disrupted rsFC on the DMN but actions of two agents on other networks diverged. The effects of two agents were robustly detected by three rsFC measures and the effect sizes were consistent across three analysis approaches. Effects of ketamine were positively correlated with the pattern of disconnectivity observed in schizophrenia patients. This correlation became significant after accounting for sedation effects of ketamine by subtracting effects of the midazolam.

## CONFLICT OF INTEREST

The authors declare no conflict of interest. PT and NJ received a research grant from Biogen, Inc., for research unrelated to the topic of this manuscript.

## Supporting information


**Appendix S1** Supporting Information

## Data Availability

Imaging data can be made available upon request.
