# Supplementary material for: Effects of ketamine and midazolam on resting state connectivity and comparison with ENIGMA connectivity deficit patterns in schizophrenia
Source: Hum Brain Mapp. 2019 Oct 21;41(3):767–78. doi: 10.1002/hbm.24838 (PMC7267897; doi:10.1002/hbm.24838)
Supplement: Supplementary file 1 — Appendix S1 Supporting Information [file HBM-41-767-s001.docx]

**Effects of Ketamine and Midazolam on resting state connectivity and comparison with ENIGMA connectivity deficit patterns in schizophrenia - Supplementary Document**

Bhim M. Adhikari^1^*, Juergen Dukart^2,5,6^*, Joerg F. Hipp^2^, Anna Forsyth^3^, Rebecca McMillan^3^, Suresh D. Muthukumaraswamy^3^, Meghann C. Ryan^1^, L. Elliot Hong^1^, Simon B. Eickhoff^5,6^, Neda Jahanshad^4^, Paul M. Thompson^4^, Laura M. Rowland^1^, Peter Kochunov^1^**

1. Maryland Psychiatric Research Center, Department of Psychiatry, University of Maryland School of Medicine, Baltimore, MD, USA
2. F. Hoffmann-La Roche, pharma Research Early Development, Roche Innovation Centre Basel, Basel, Switzerland
3. School of Pharmacy, Faculty of Medical and Health Sciences, The University of Auckland, Auckland, New Zealand
4. Imaging Genetics Center, Mark & Mary Stevens Neuroimaging & Informatics Institute, Keck School of Medicine, University of Southern California, Marina del Rey, CA, USA
5. Institute of Neuroscience and Medicine, Brain & Behaviour (INM-7), Research Centre Jülich, Jülich, Germany
6. Institute of Systems Neuroscience, Medical Faculty, Heinrich Heine University Düsseldorf, Düsseldorf, Germany

*BA and JD contributed equally to this manuscript.

**Corresponding author: [pkochunov@som.umaryland.edu](mailto:pkochunov@som.umaryland.edu)

Maryland Psychiatry Research Center, Department of Psychiatry

University of Maryland School of Medicine, Baltimore, MD, USA

Phone: (410) 402-6110; Fax: (410) 402-6778

**Physiological data acquisition**

During each scan, participants wore a respiration belt and a pulse plethsmyograph on the left index finger (Biopac, USA). Participants were fitted with a nasal cannula to measure end-tidal oxygen and carbon dioxide levels. For safety monitoring, an additional pulse oximeter was placed on the middle finger of the left hand to measure heart rate and blood oxygen saturation (Nonin, USA). All these signals were recorded on a Biopac MP150 (California: USA) system. A blood pressure cuff was placed on participant’s right arm for periodic (between scan) measurements of blood pressure.

**Resting state functional MRI (rsfMRI) data processing and analysis**

The ENIGMA resting state analysis pipeline is a single-modality analysis pipeline (Adhikari et al., 2018c), an extension of the conventional AFNI rsfMRI pipeline (detail description in (Adhikari et al., 2018a)) **(Figure S1).** The first step in the analysis pipeline is the implementation of the principal components analysis (PCA)-based denoising (Veraart et al., 2016a; Veraart et al., 2016b) approach to improve signal-to-noise ratio (SNR) and temporal SNR (tSNR). This denoising approach, called MPPCA, neither alters the spatial resolution of the image nor introduces the additional partial volume effects (Veraart et al., 2016b). SNR improvement using spatial smoothing reduces in spatial specificity, and thus complicates localization of effects in the rsfMRI/fMRI images. Moreover, the noise-maps produced by MPPCA approach provide valuable information for quality control as deviations from the expected uniform or slowly varying in space pattern of thermal noise may indicate problems with the coil or other scanner hardware.

In the next step, a transformation is computed registering the base volume to the ENIGMA EPI template, derived from ~1,100 datasets collected across 22 sites (Adhikari et al., 2018c). This atlas is used as a common anatomical spatial reference frame and for regression of the global signal. Head motion correction is performed by registering each functional volume to the volume with the minimum outlier fraction, where each transformation is concatenated with the transformation to standard space, to avoid unnecessary interpolation. Nuisance variables such as the linear trend, 6 motion parameters, their 6 temporal derivatives, and time courses from the local white matter and cerebrospinal fluid from lateral ventricles were modeled using multiple linear regression analysis, which were then removed as regressors of no interest. Time points with excessive motion (> 0.2 mm), estimated as the magnitude of displacement from one time point to the next, including neighboring time points and outlier voxels fraction (> 0.1) were censored from statistical analysis. Images were spatially normalized to the ENIGMA EPI template in Montreal Neurological Institute standard space for group analysis.


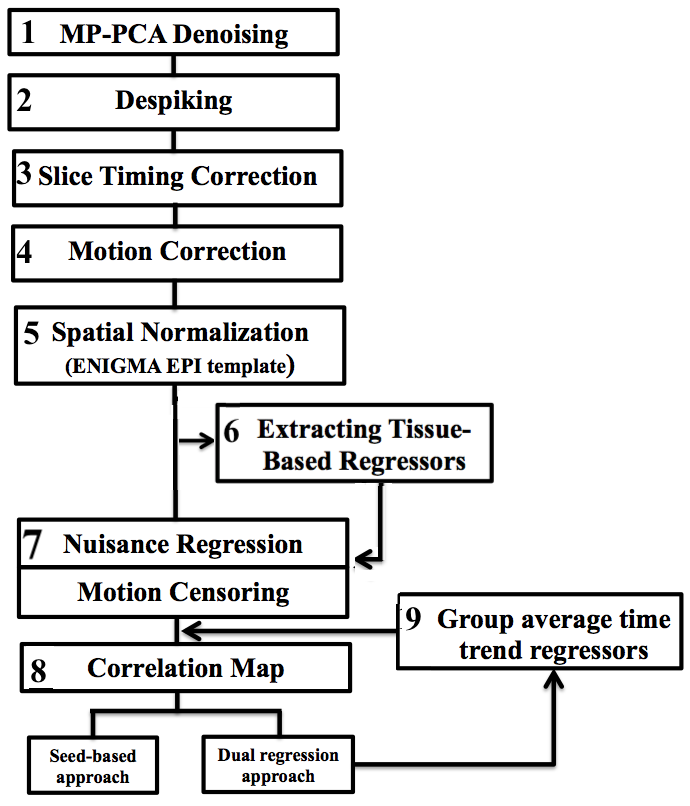


**Figure S1**. Flowchart of an ENIGMA rsfMRI analysis pipeline (Adhikari et al., 2018b).

**Functional connectivity analysis**

Resting state network templates were defined based on the probabilistic regions of interest (ROIs) from independent component analysis of the BrainMap activation database and resting fMRI dataset (Smith et al., 2009). We defined the binary masks of the resting state template regions from auditory network (AN), attention network (AttN), default mode network (DMN), executive-control network (ECN), fronto-parietal network (FPN), salience network (SN), sensorimotor network (SMN), and visual network (VN) (**Figure S2**) (Adhikari et al., 2018a). The colored regions in **Figure S2** represent the ‘seeds’ for the functional connectivity analysis.

**
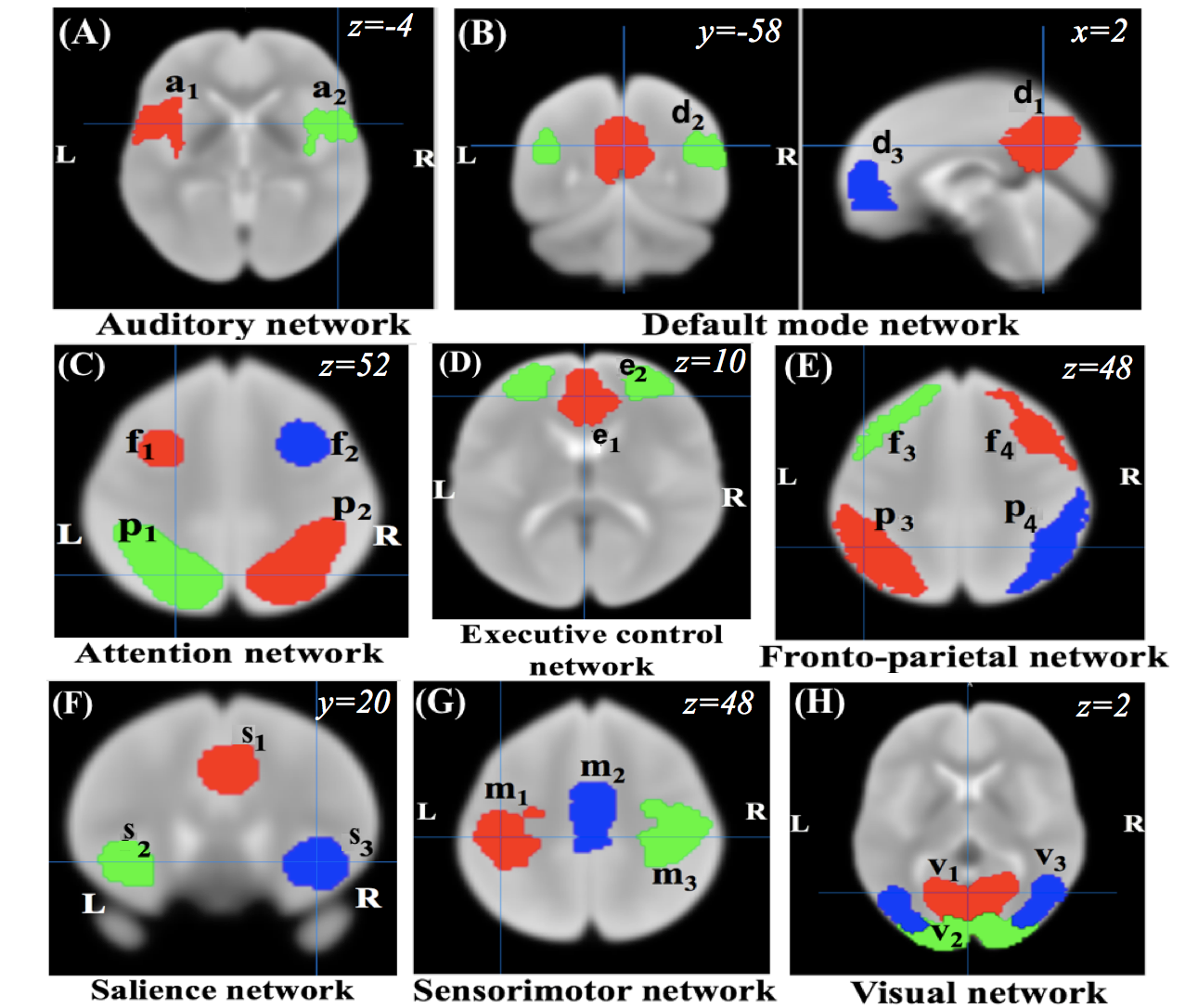
**

**Figure S2.** Resting state network template ROIs based on the BrainMap activation database (Smith et al., 2009). Here, L, left, R, right, in (A) a_1_/a_2_, left/right primary and association auditory cortices; in (B) d_1_, posterior cingulate/precuneus, d_2_, bilateral temporal-parietal regions, and d_3_, ventromedial frontal cortex; in (C) f_1_/f_2_ , left/right middle frontal gyrus and p_1_/p_2_, left/right superior parietal lobule; in (D) e_1_, anterior cingulate cortex and e_2_, bilateral medial frontal gyrus; in (E) f_3_/f_4_, left/right inferior frontal gyrus and p_3_/p_4_, left/right inferior parietal lobule; in (F) s_1_, anterior cingulate cortex and s_2_/s_3_, left/right insula; in (G) m_1_/m_3_, left/right motor area and m_2_, supplementary motor area; and in (H) v_1_, medial visual areas, v_2_, occipital visual areas, and v_3_, lateral visual areas. The coordinates of the given slice are also provided.

Mean time series were extracted from the seed regions of each network and connectivity maps corresponding to each seed region were obtained by assessing correlations along the time series for different regions. Next, Fisher’s *r*-to-*z* transformations were applied to obtain a normal distribution. We calculated seed-based (node-based) functional connectivity values between seed regions in each network. Furthermore, we performed dual regression analysis for these all network template ROIs and calculated the functional connectivity measures.

We developed a modification of the dual regression approaches as implemented in the FSL software (Beckmann et al., 2009; Filippini et al., 2009). FSL’s dual regression approach uses the spatial maps from the group-average analysis to generate subject-specific spatial maps, and extract subject-specific time series. For each subject, the group-average set of spatial maps is regressed into the subject's 4D space-time dataset. This results in a set of subject-specific time series, one per group-level spatial map. Next, those time series are regressed into the same 4D dataset, resulting in a set of subject-specific spatial maps, one per group-level spatial map.

We have used the spatial maps template of resting networks from independent component analysis-based analyses of the BrainMap database. Following the preprocessing steps, we computed the average time series for the ROIs included in the given network template for each subject. We obtained the average time series from all subjects that represent the group’s time-trend for the corresponding network. We regressed out this trend from individual subject’s data. This was performed by including the group average trend for a network as an additional regressor appended to the matrix. This was achieved by re-running the step 9 (**Figure S1**) because the group average trend is not expected to be orthogonal to the other nuisance regressors for any given subject (e.g., motion parameters). After regressing out the effects of the group average trend, we calculated correlation brain maps corresponding to each seed region for a given network, performed Fisher’s *r*-to-*z* transformations and extracted FC values between seed regions in each network template. The dual regression approach provides a statistical inference on how the individual subjects differ from the group average.

Results of the dual regression analysis were very similar to that for the seed-based analyses (**Figure S3, Table S1**). Significantly reduced rsFC strength was observed for all AN (*p* < 0.01) and SN (*p* < 10^-4^) connections and the connections: posterior cingulate/precuneus to bilateral temporo-parietal region and vice versa, posterior cingulate/precuneus to ventromedial frontal cortex and vice versa for DMN for ketamine administration. Following the administration


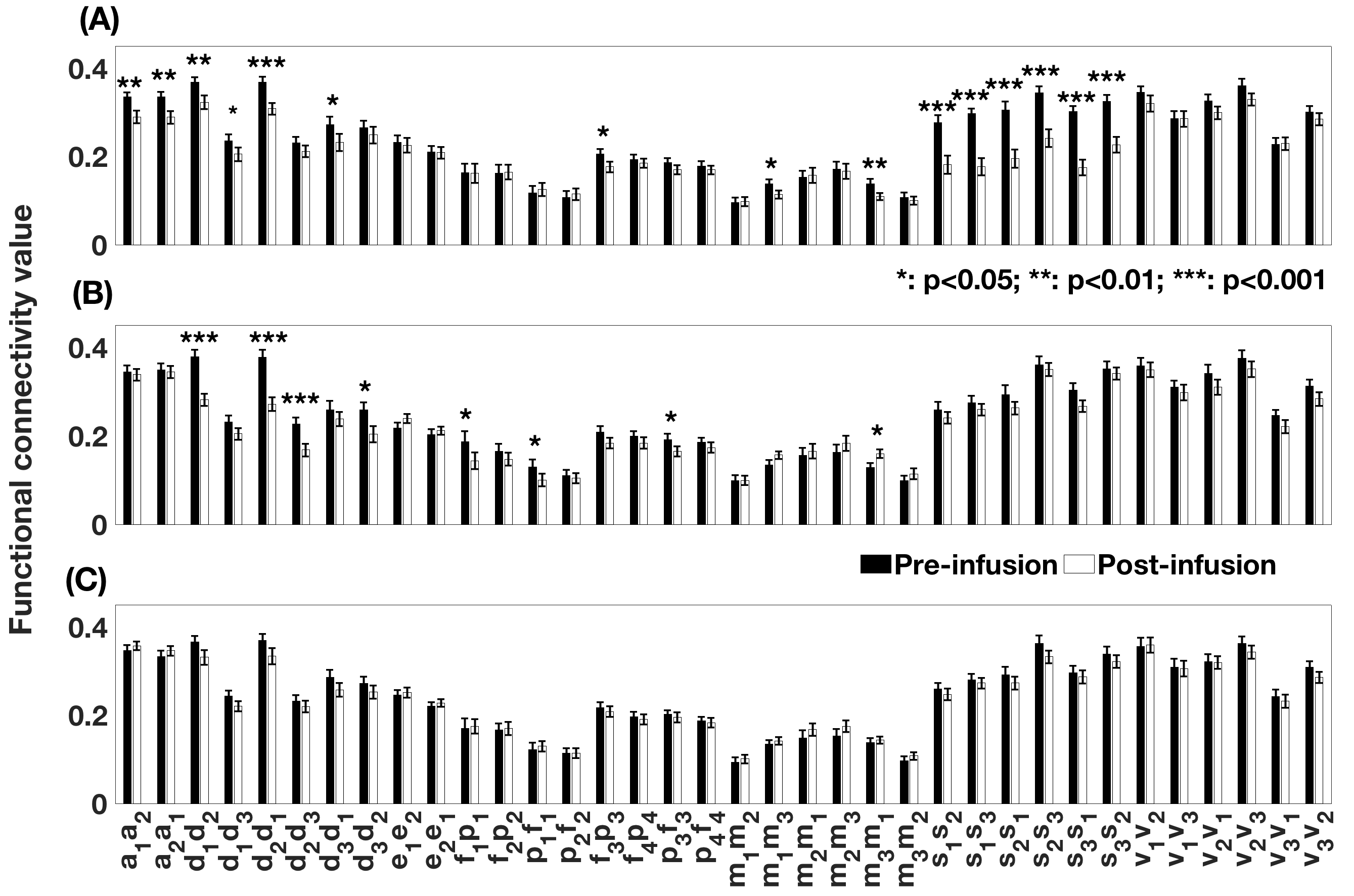


**Figure S3**. Comparison of the resting state functional connectivity strengths, post-infusion relative to pre-infusion, for (A) ketamine (B) midazolam, and (C) placebo in dual regression analysis. The functional connections are defined using the regions, based on **Figure S2**.

of midazolam, significant reductions in rsFC were observed in the DMN (connections: from posterior cingulate/precuneus to bilateral temporo-parietal region and vice versa and bilateral temporo-parietal region to ventromedial frontal cortex and vice versa), the AttN (connection: from left middle frontal gyrus to left superior parietal lobule), and the FPN (connection: from left inferior parietal lobule to left inferior frontal gyrus). But for a SMN connection; from right motor cortex to left motor cortex, showed a significant increase in rsFC measures. No significant reduction in rsFC was observed in placebo.

The comparison of rsFC measures between after infusion minus no infusion of the drugs versus with placebo minus no placebo condition are shown in **Figure S4.** We found a significant reduction in rsFC strengths in SN (*p* < 0.001), AN (*p* < 0.01), and the connections from left motor cortex to right motor cortex and vice-versa in SMN (*p* < 0.05) for ketamine. In case of midazolam, significant reductions in rsFC strengths were observed in the DMN (from the posterior cingulate/precuneus to bilateral temporal-parietal region and vice versa) and the AttN (from left middle frontal gyrus to left superior parietal lobule and vice-versa) connections (*p* < 0.01).


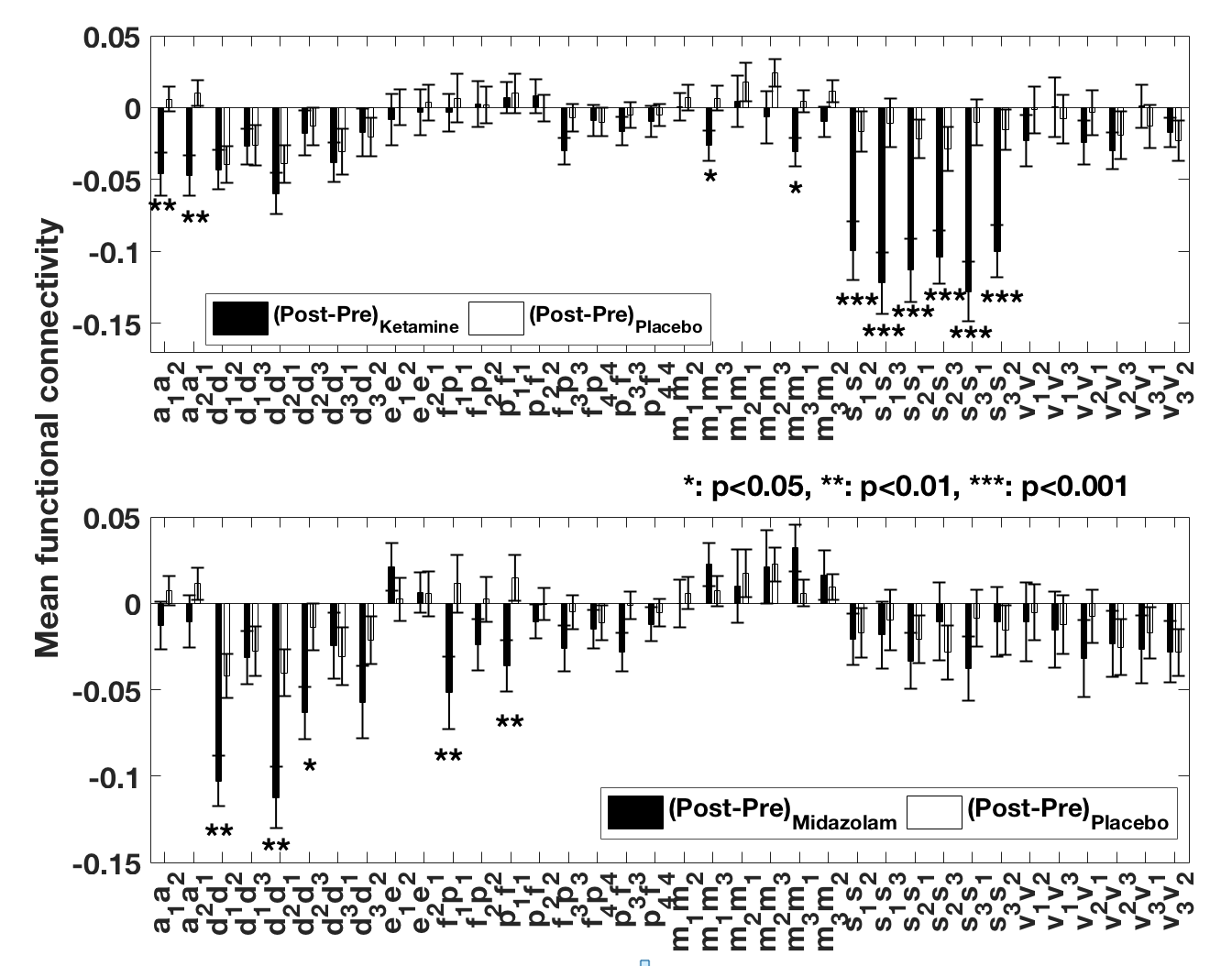


**Figure S4.** Comparison of the rsFC measures, derived from RSNs using seed-based analysis approach, for administration of drugs versus placebo condition ((Post-Pre)_Ketamine/Midazolam_ versus (Post-Pre)_placebo_). Plot in upper panel is for ketamine and lower panel is for midazolam. The functional connections are defined using the regions, based on **Figure S2**.

The ReHo scores between administration of drugs versus placebo condition (**Figure S5**) were found significantly reduced for all RSNs ROIs for ketamine administration (*p* < 0.01). The midazolam effects were observed for the DMN, FPN, ECN ROIs and frontal regions of AttN, without significant changes on the AN, SN, SMN, and VN regions.

**
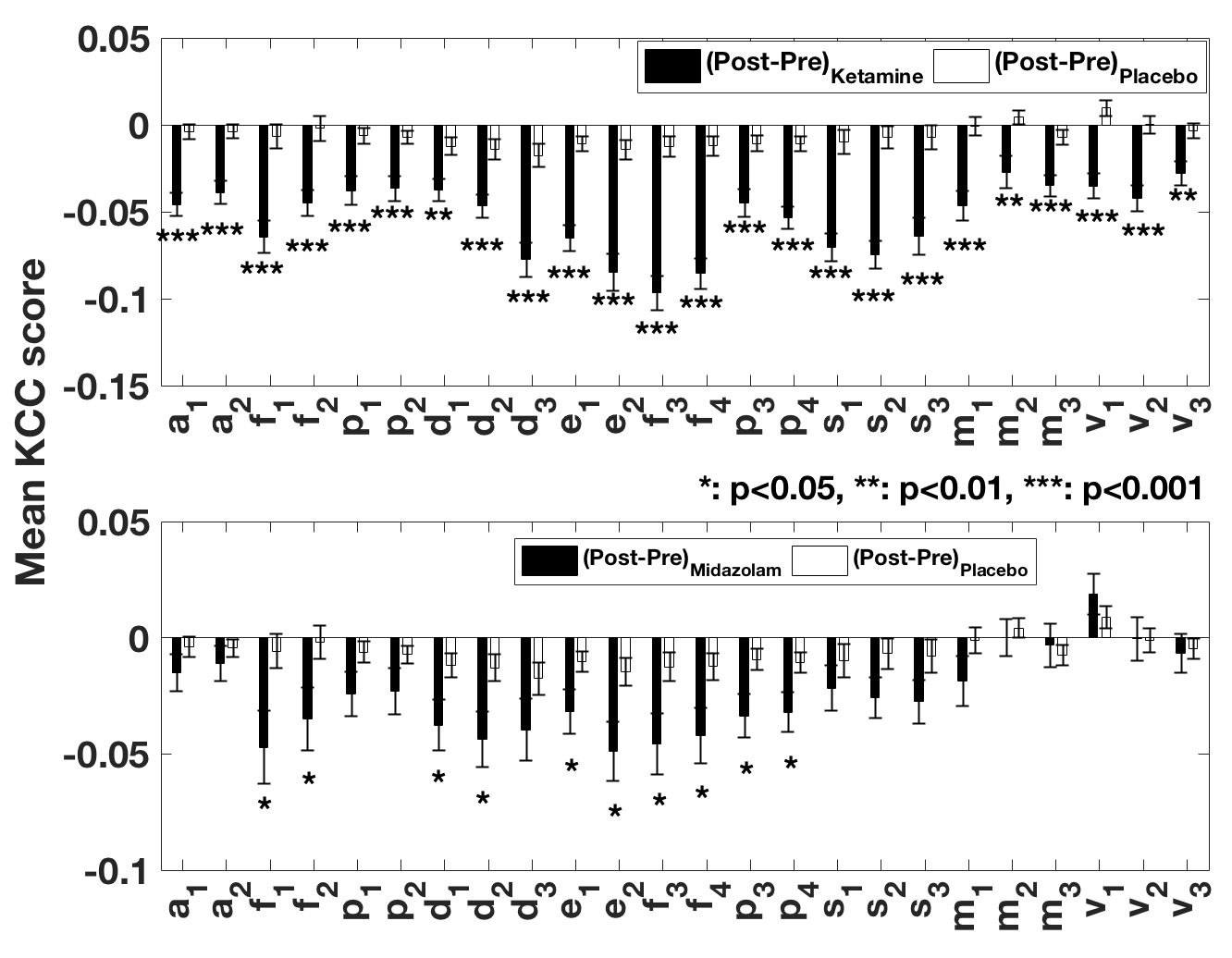
**

**Figure S5.** Comparison of the regional homogeneity (Kendall’s coefficient of concordance, KCC) scores derived from RSNs ROIs for the administration of drugs versus placebo condition [(Post-Pre)_Ketamine/Midazolam_ versus (Post-Pre)_placebo)_]. Upper panel plot is for ketamine and lower panel plot is for midazolam.

**Table S1**. Statistical measures (*p*-value and *t*-value) for the comparison between functional connectivity measures derived from resting state networks (RSNs) using dual regression analysis approach for ketamine and midazolam administration and placebo condition (post-infusion versus pre-infusion). Bolded figures are significant for *p* < 0.05. Regions are based on **Figure S2**.

| Network | Connections | Ketamine | Midazolam | Placebo |
| --- | --- | --- | --- | --- |
|  |  | *p* (*t*) | *p* (*t*) | *p* (*t*) |
| Auditory network (AN) | Auditory cortices L – Auditory cortices R | **8.0×10^-3^ (-2.84)** | 0.64 (-0.48) | 0.21 (1.29) |
|  | Auditory cortices R – Auditory cortices L | **1.3×10^-3^ (-3.74) *** | 0.77 (-0.29) | 0.13 (1.56) |
| Attention network (AttN) | Middle FG L – SPL L | 0.89 (-0.14) | **3.7×10^-2^ (-2.19)** | 0.79 (0.26) |
|  | SPL L – Middle FG L | 0.49 (0.70) | 0.06 (-1.96) | 0.58 (0.56) |
|  | Middle FG R – SPL R | 0.88 (0.15) | 0.21 (-1.30) | 0.78 (0.28) |
|  | SPL R – middle FG R | 0.54 (0.63) | 0.57 (-0.58) | 0.99 (0.00) |
| Default mode network (DMN) | PCC/precuneus – Bilateral temporal-parietal region | **1.2×10^-3^ (-3.77) *** | **1.0×10^-6^ (-6.28) *** | 0.19 (-1.29) |
|  | PCC/precuneus – vmFC | **1.8×10^-2^ (-2.51)** | 0.100 (-1.68) | 0.12 (-1.60) |
|  | Bilateral temporal-parietal region – PCC/precuneus | **3.0×10^-4^ (-4.08) *** | **3.3×10^-6^ (-5.84) *** | 0.15 (-1.45) |
|  | Bilateral temporal-parietal region – vmFC | 0.25 (-1.17) | **8.6×10^-4^ (-3.75) *** | 0.38 (-0.88) |
|  | vmFC – PCC/precuneus | **6.0×10^-3^ (-2.98) *** | 0.30 (-1.05) | 0.10 (-1.69) |
|  | vmFC– Bilateral temporal-parietal region | 0.32 (-1.02) | **1.9×10^-2^ (-2.49)** | 0.18 (-1.36) |
| Executive control network (ECN) | ACC – Bilateral medial FG | 0.70 (-0.39) | 0.11 (1.66) | 0.63 (0.49) |
|  | Bilateral medial FG – ACC | 0.95 (-0.07) | 0.44 (0.79) | 0.58 (0.56) |
| Fronto-parietal network (FPN) | Frontal area L – Parietal area L | **2.0×10^-3^ (-3.39)** | 0.07 (-1.90) | 0.33 (-1.00) |
|  | Parietal area L – Frontal area L | 0.13 (-1.58) | **2.1×10^-2^ (-2.46)** | 0.44 (-0.78) |
|  | Frontal area R – Parietal area R | 0.44 (-0.79) | 0.20 (-1.33) | 0.53 (-0.63) |
|  | Parietal area R – Frontal area R | 0.40 (-0.85) | 0.24 (-1.20) | 0.60 (-0.53) |
| Salience network (SN) | ACC – INS L | **3.7×10^-5^ (-4.90) *** | 0.25 (-1.19) | 0.43 (-0.81) |
|  | ACC – INS R | **1.8×10^-6^ (-6.00) *** | 0.43 (-0.81) | 0.69 (-0.40) |
|  | INS L – ACC | **1.5×10^-5^ (-5.22) *** | 0.08 (-1.81) | 0.18 (-1.39) |
|  | INS L – INS R | **2.5×10^-6^ (-5.89) *** | 0.66 (-0.44) | 0.06 (-1.97) |
|  | INS R – ACC | **6.2×10^-7^ (-6.40) *** | 0.06 (-1.99) | 0.53 (-0.63) |
|  | INS R – INS L | **7.3×10^-6^ (-5.49) *** | 0.59 (-0.54) | 0.20 (-1.31) |
| Sensorimotor network (SMN) | Motor area L – SMA | 0.47 (-0.73) | 0.95 (0.07) | 0.40 (0.86) |
|  | Motor area L – Motor area R | **1.6×10^-2^ (-2.56)** | 0.07 (1.89) | 0.41 (0.83) |
|  | SMA – Motor area L | 0.49 (-0.70) | 0.63 (0.49) | 0.14 (1.51) |
|  | SMA – Motor area R | 0.54 (0.62) | 0.32 (1.01) | 0.06 (1.91) |
|  | Motor area R – motor area L | **6.0×10^-3^ (-2.97)** | **2.3×10^-2^ (2.42)** | 0.46 (0.75) |
|  | Motor area R – SMA | 0.40 (-0.85) | 0.28 (1.09) | 0.14 (1.51) |
| Visual network (VN) | Medial visual areas–Occipital visual areas | 0.16 (-1.44) | 0.71 (-0.38) | 0.84 (0.20) |
|  | Medial visual areas – Lateral visual areas | 0.99 (0.00) | 0.57 (-0.57) | 0.85 (-0.20) |
|  | Occipital visual areas–Medial visual areas | 0.09 (-1.75) | 0.16 (-1.44) | 0.90 (-0.12) |
|  | Occipital visual areas–Lateral visual areas | 0.13 (-1.58) | 0.19 (-1.33) | 0.23 (-1.24) |
|  | Lateral visual areas – Medial visual areas | 0.92 (0.10) | 0.22 (-1.27) | 0.47 (-0.74) |
|  | Lateral visual areas–Occipital visual areas | 0.08 (-1.83) | 0.12 (-1.60) | 0.10 (-1.70) |

Abbreviations: L, left, R, right, FG, frontal gyrus, SPL, superior parietal lobule, PCC, posterior cingulate cortex, vmFC, ventromedial frontal cortex, ACC, anterior cingulate cortex, INS, insula, SMA, supplementary motor area. * Values those are significant after correction for multiple comparisons.

**Table S2**. Effect sizes, *Cohen’s d* ± *s. e.* (*p*-value, in the parentheses) for the functional connectivity (FC) values obtained from the seed-based analysis, dual regression analysis, and regional homogeneity (ReHo) methods for ketamine, midazolam and placebo from resting state networks.

| Analysis | Networks | Ketamine | Midazolam | Placebo |
| --- | --- | --- | --- | --- |
| Seed-based | AN | **-0.67 ± 0.26 (0.04)** | -0.15 ± 0.26 (0.36) | 0.18 ± 0.26 (0.32) |
|  | lAttN | -0.18 ± 0.26 (0.32) | -0.43 ± 0.26 (0.15) | 0.06 ± 0.26 (0.43) |
|  | rAttN | -0.16 ± 0.26 (0.34) | -0.21 ± 0.26 (0.30) | 0.03 ± 0.26 (0.47) |
|  | DMN | **-0.63 ± 0.26 (0.05)** | **-0.77 ± 0.27 (0.03)** | -0.35 ± 0.26 (0.17) |
|  | ECN | -0.07 ± 0.26 (0.43) | 0.21 ± 0.26 (0.31) | 0.10 ± 0.26 (0.39) |
|  | lFPN | -0.37 ± 0.26 (0.18) | -0.39 ± 0.26 (0.18) | -0.12 ± 0.26 (0.38) |
|  | rFPN | -0.16 ± 0.26 (0.35) | -0.22 ± 0.26 (0.30) | -0.10 ± 0.26 (0.40) |
|  | SMN | -0.21 ± 0.26 (0.30) | 0.26 ± 0.26 (0.27) | 0.17 ± 0.26 (0.33) |
|  | SN | **-1.13 ± 0.28 (4×10^-3^)** | -0.26 ± 0.26 (0.27) | -0.15 ± 0.26 (0.35) |
|  | VN | 0.03 ± 0.26 (0.47) | -0.26 ± 0.26 (0.27) | -0.10 ± 0.26 (0.40) |
| Dual regression | AN | **-0.68 ± 0.27 (0.04)** | -0.07 ± 0.26 (0.43) | 0.19 ± 0.26 (0.46) |
|  | lAttN | -0.20 ± 0.26 (0.31) | -0.37 ±0 .26 (0.19) | 0.07 ± 0.26 (0.32) |
|  | rAttN | -0.15 ± 0.26 (0.41) | -0.15 ± 0.26 (0.36) | 0.02 ± 0.26 (0.44) |
|  | DMN | **-0.64 ± 0.26 (0.05)** | **-0.72 ± 0 .27 (.04)** | -0.31 ± 0.26 (0.22) |
|  | ECN | -0.04 ± 0.26 (0.45) | 0.25 ± 0.26 (0.28) | 0.12 ± 0.26 (0.38) |
|  | lFPN | -0.38 ± 0.26 (0.19) | -0.38 ± 0.26 (0.18) | -0.13 ± 0.26 (0.36) |
|  | rFPN | -0.15 ± 0.26 (0.35) | -0.23 ± 0.26 (0.29) | -0.09 ± 0.26 (0.42) |
|  | SMN | -0.20 ± 0.26 (0.31) | 0.27 ± 0.26 (0.26) | 0.18 ± 0.26 (0.32) |
|  | SN | **-1.18 ± 0.28 (3×10^-3^)** | -0.24 ± 0.26 (0.28) | -0.18 ± 0.26 (0.32) |
|  | VN | 0.04 ± 0.26 (0.46) | -0.25 ± 0.26 (0.27) | -0.11 ± 0.26 (0.38) |
| Regional  homogeneity | AN | **-1.46 ± 0.29 (5×10^-4^)** | -0.27 ± 0.26 (0.26) | -0.04 ± 0.26 (0.46) |
|  | lAttN | **-0.86 ± 0.27 (0.02)** | **-0.77 ± 0.27 (0.03)** | -0.16 ± 0.26 (0.34) |
|  | rAttN | **-1.13 ± 0.28 (4×10^-3^)** | -0.63 ± 0.26 (0.07) | -0.31 ± 0.26 (0.21) |
|  | DMN | **-1.54 ± 0.29 (3×10^-4^)** | -0.49 ± 0.26 (0.12) | -0.39 ± 0.26 (0.15) |
|  | ECN | **-1.53 ± 0.29 (3×10^-3^)** | 0.06 ± 0.26 (0.44) | -0.20 ± 0.26 (0.30) |
|  | lFPN | **-1.16 ± 0.28 (3×10^-3^)** | -0.58 ± 0.26 (0.08) | -0.33 ± 0.26 (0.20) |
|  | rFPN | **-1.28 ± 0.28 (2×10^-3^)** | **-0.68 ± 0.27 (0.04)** | -0.30 ± 0.26 (0.22) |
|  | SMN | **-1.16 ± 0.28 (3×10^-3^)** | -0.14 ± 0.26 (0.37) | 0.07 ± 0.26 (0.42) |
|  | SN | **-1.70 ± 0.30 (1×10^-4^)** | -0.63 ± 0.26 (0.07) | -0.09 ± 0.26 (0.41) |
|  | VN | **-1.04 ± 0.28 (7×10^-3^)** | **-0.69 ± 0.27 (0.04)** | -0.15 ± 0.26 (0.35) |

Bolded figures are significant for *p* < 0.05.

**Table S3**. Mega-analytical statistics comparing groups of schizophrenia patients and healthy controls using functional connectivity measures from resting state networks using seed-based analysis approach. Effect size values (in **bold font**) survived for multiple comparison corrections (Bonferroni correction, *p* < 0.05).

|  |  | Seed-based analysis approach | | | |
| --- | --- | --- | --- | --- | --- |
| Network | Connections | Effect size | 95%-Confident interval | *z*-value | *p*-value |
| AN | a_1_a_2_ | **-0.54** | [-0.70; -0.37] | -6.35 | 2.2×10^-10^ |
|  | a_2_a_1_ | **-0.51** | [-0.68; -0.34] | -6.04 | 1.5×10^-9^ |
| AttN | f_1_p_1_ | **-0.35** | [-0.51; -0.18] | -4.15 | 3.3×10^-5^ |
|  | p_1_f_1_ | **-0.35** | [-0.51; -0.19] | -4.19 | 2.8×10^-5^ |
|  | f_2_p_2_ | -0.25 | [-0.42; -0.09] | -3.03 | 0.0025 |
|  | p_2_f_2_ | **-0.28** | [-0.44; -0.11] | -3.33 | 9.0×10^-4^ |
| DMN | d_1_d_2_ | -0.25 | [-0.41; -0.08] | -2.97 | 0.0030 |
|  | d_2_d_3_ | -0.23 | [-0.39; -0.06] | -2.71 | 0.0067 |
|  | d_3_d_1_ | -0.27 | [-0.43; -0.10] | -3.18 | 0.0015 |
|  | d_2_d_1_ | -0.21 | [-0.37; -0.05] | -2.51 | 0.0122 |
|  | d_3_d_2_ | -0.19 | [-0.36; -0.03] | -2.34 | 0.0192 |
|  | d_1_d_3_ | **-0.27** | [-0.44; -0.11] | -3.29 | 1.0×10^-3^ |
| ECN | e_1_e_2_ | **-0.37** | [-0.54; -0.21] | -4.46 | 8.1×10^-6^ |
|  | e_2_e_1_ | -0.21 | [-0.38; -0.05] | -2.57 | 0.0101 |
| FPN | f_3_p_3_ | -0.24 | [-0.40; -0.08] | -2.86 | 0.0042 |
|  | p_3_f_3_ | -0.20 | [-0.36; -0.03] | -2.37 | 0.0178 |
|  | f_4_p_4_ | -0.24 | [-0.41; -0.08] | -2.94 | 0.0033 |
|  | p_4_f_4_ | -0.22 | [-0.38; -0.06] | -2.64 | 0.0084 |
| SN | s_1_s_2_ | **-0.46** | [-0.62; -0.29] | -5.46 | 4.7×10^-8^ |
|  | s_2_s_3_ | **-0.40** | [-0.56; -0.23] | -4.75 | 2.1×10^-6^ |
|  | s_3_s_1_ | **-0.42** | [-0.58; -0.25] | -4.97 | 6.8×10^-7^ |
|  | s_2_s_1_ | **-0.49** | [-0.65; -0.32] | -5.76 | 8.2×10^-9^ |
|  | s_3_s_2_ | **-0.39** | [-0.55; -0.22] | -4.63 | 3.6×10^-6^ |
|  | s_1_s_3_ | **-0.43** | [-0.60; -0.27] | -5.15 | 2.6×10^-7^ |
| SMN | m_1_m_2_ | **-0.29** | [-0.45; -0.12] | -3.40 | 7.0×10^-4^ |
|  | m_2_m_3_ | -0.26 | [-0.42; -0.10] | -3.11 | 0.0019 |
|  | m_3_m_1_ | -**0.35** | [-0.52; -0.19] | -4.21 | 2.6×10^-5^ |
|  | m_2_m_1_ | **-0.35** | [-0.51; -0.18] | -4.16 | 3.1×10^-5^ |
|  | m_3_m_2_ | -0.21 | [-0.38; -0.05] | -2.55 | 0.0107 |
|  | m_1_m_3_ | **-0.33** | [-0.49; -0.16] | -3.91 | 9.3×10^-5^ |
| VN | v_1_v_2_ | **-0.27** | [-0.44; -0.11] | -3.27 | 1.1×10^-3^ |
|  | v_2_v_3_ | **-0.29** | [-0.46; -0.13] | -3.51 | 5.0×10^-4^ |
|  | v_3_v_1_ | -0.20 | [-0.37; -0.04] | -2.45 | 0.0144 |
|  | v_2_v_1_ | -0.21 | [-0.38; -0.05] | -2.56 | 0.0106 |
|  | v_3_v_2_ | **-0.29** | [-0.45; -0.13] | -3.46 | 5.0×10^-4^ |
|  | v_1_v_3_ | -0.25 | [-0.42; -0.09] | -3.04 | 0.0024 |
| Mean effect size | | -0.31 | [-0.33; -0.28] | -19.5 | <1.0×10^-4^ |

Abbreviations: AN, auditory network, a_1_/a_2,_ left/right primary and association auditory cortices; AttN, attention network, f_1_/f_2_, left/right middle frontal gyrus, and p_1_/p_2_, left/right superior parietal lobule; DMN, default mode network, d_1_, posterior cingulate/precuneus, d_2_, bilateral temporal-parietal regions and, d_3_, ventromedial frontal cortex; ECN, executive control network, e_1_, anterior cingulate cortex and e_2_, bilateral medial frontal gyrus; FPN, fronto-parietal network, f_3_/f_4_, left/right frontal area (inferior frontal gyrus) and p_3_/p_4_, left/right parietal area (inferior parietal lobule); SN, salience network, s_1_, anterior cingulate cortex and s_2_/s_3_, left/right insula; SMN, sensorimotor network, m_1_/m_3_, left/right motor area and m_2_, supplementary motor area; and VN, visual network, v_1_, medial visual areas, v_2_, occipital visual areas, and v_3_, lateral visual areas.

**References**

Adhikari, B.M., Jahanshad, N., Shukla, D., Glahn, D.C., Blangero, J., Reynolds, R.C., . . . Kochunov, P. (2018a) Comparison of heritability estimates on resting state fMRI connectivity phenotypes using the ENIGMA analysis pipeline. Human Brain Mapping, 39:4893-4902.

Adhikari, B.M., Jahanshad, N., Shukla, D.K., Glahn, D.C., Blangero, J., Reynolds, R.C., . . . Kochunov, P. (2018b) Heritability estimates on resting state fMRI data using ENIGMA analysis pipeline. Pac Symp Biocomput, 23:307-318.

Adhikari, B.M., Jahanshad, N., Shukla, D.K., Turner, J.A., Grotegerd, D., Dannlowski, U., . . . Kochunov, P. (2018c) A resting state fMRI analysis pipeline for pooling inference across diverse cohorts: An ENIGMA rs-fMRI Protocol. Brain Imaging and Behavior, 13: 1453-1467, <https://doi.org/10.1007/s11682-018-9941-x>.

Beckmann, C.F., Mackay, C.E., Filippini, N., & Smith, S.M. (2009) Group comparison of resting-state FMRI data using multi-subject ICA and dual regression. OHBM, 2009. NeuroImage, 47 (Suppl 1):S148.

Filippini, N., MacIntosh, B.J., Hough, M.G., Goodwin, G.M., Frisoni, G.B., Smith, S.M., . . . Mackay, C.E. (2009) Distinct patterns of brain activity in young carriers of the APOE-ε4 allele. Proc. Natl. Acad. Sci. U.S.A., 106:7209-7214.

Smith, S.M., Fox, P.T., Miller, K.L., Glahn, D.C., Fox, P.M., Mackay, C.E., . . . Beckmann, C.F. (2009) Correspondence of the brain’s functional architecture during activation and rest. Proc. Natl. Acad. Sci. USA, 106:13040-13045.

Veraart, J., Fieremans, E., & Novikov, D.S. (2016a) Diffusion MRI noise mapping using random matrix theory. Magn Reson Med, 76:1582-1593.

Veraart, J., Novikov, D.S., Christiaens, D., Ades-Aron, B., Sijbers, J., & Fieremans, E. (2016b) Denoising of diffusion MRI using random matrix theory. Neuroimage, 142:394-406.
